# Distinct Responses to Menin Inhibition and Synergy with DOT1L Inhibition in *KMT2A*-Rearranged Acute Lymphoblastic and Myeloid Leukemia

**DOI:** 10.3390/ijms25116020

**Published:** 2024-05-30

**Authors:** Fabienne R. S. Adriaanse, Pauline Schneider, Susan T. C. J. M. Arentsen-Peters, Ana M. Neves da Fonseca, Janine Stutterheim, Rob Pieters, C. Michel Zwaan, Ronald W. Stam

**Affiliations:** 1 Princess Maxima Center for Pediatric Oncology, 3584 CS Utrecht, The Netherlands; 2 Department of Pediatric Oncology, Erasmus MC-Sophia’s Children’s Hospital, 3015 CN Rotterdam, The Netherlands

**Keywords:** *KMT2A*-rearranged, pediatric, AML, infant, ALL, revumenib, menin, synergy, DOT1L, pinometostat

## Abstract

Pediatric acute myeloid leukemia (AML) and acute lymphoblastic leukemia (ALL) exhibit favorable survival rates. However, for AML and ALL patients carrying *KMT2A* gene translocations clinical outcome remains unsatisfactory. Key players in KMT2A-fusion-driven leukemogenesis include menin and DOT1L. Recently, menin inhibitors like revumenib have garnered attention for their potential therapeutic efficacy in treating *KMT2A*-rearranged acute leukemias. However, resistance to menin inhibition poses challenges, and identifying which patients would benefit from revumenib treatment is crucial. Here, we investigated the in vitro response to revumenib in *KMT2A*-rearranged ALL and AML. While ALL samples show rapid, dose-dependent induction of leukemic cell death, AML responses are much slower and promote myeloid differentiation. Furthermore, we reveal that acquired resistance to revumenib in *KMT2A*-rearranged ALL cells can occur either through the acquisition of *MEN1* mutations or independently of mutations in *MEN1*. Finally, we demonstrate significant synergy between revumenib and the DOT1L inhibitor pinometostat in *KMT2A*-rearranged ALL, suggesting that such drug combinations represent a potent therapeutic strategy for these patients. Collectively, our findings underscore the complexity of resistance mechanisms and advocate for precise patient stratification to optimize the use of menin inhibitors in *KMT2A*-rearranged acute leukemia.

## 1. Introduction

Nowadays, the overall survival rates for pediatric acute myeloid leukemia (AML) and acute lymphoblastic leukemia (ALL) are quite favorable, ranging between 70 and 94%, respectively [1,2,3]. However, clinical outcomes for ALL and AML patients harboring a chromosomal translocation involving the *lysine methyl transferase 2A* (*KMT2A*) gene remain unsatisfactory, with a high risk of treatment failure and event-free survival rates of only 20–40% when treated with standard combination chemotherapy [4,5,6,7] in AML depending on the *KMT2A* translocation [5].

During *KMT2A* translocations, the N-terminus of the *KMT2A* gene fuses to the C-terminus of one of its translocation partner genes, which most commonly include *AFF1*, *MLLT1*, *MLLT3,* and *MLLT10* [8]. The resulting *KMT2A* fusion genes give rise to chimeric fusion proteins representing strong oncogenic drivers of leukemia development. The leukemogenic activity of these KMT2A fusion proteins largely depends on their direct interactions with the scaffold protein menin and the N-terminus of KMT2A, and the recruitment of the histone H3K79 methyltransferase DOT1L by the C-terminus of the fusion [9,10,11,12,13]. As such, menin and DOT1L are recognized as critical players in *KMT2A*-fusion-driven gene expression, including the expression of *HOXA* genes, *MEIS1*, *CDK6*, and *MEF2C* [14,15,16,17,18]. Consequently, there has been great interest in preventing menin from binding to KMT2A protein complexes using small molecules, as several studies showed promising therapeutic potential for *KMT2A*-rearranged acute leukemia [19,20,21,22,23,24]. In recent years, novel menin inhibitors were developed with enhanced selectivity, potency, improved pharmaceutical properties, and tremendous anti-leukemic activity. In patient-derived xenograft (PDX) mouse models of *KMT2A*-rearranged acute leukemia, these compounds induced (near) complete remissions even when administered as single agents [25,26]. Moreover, in vitro and in vivo studies revealed potential advantages of co-treatment with menin inhibitors such as SNDX-50469 (VTP50469), revumenib (SNDX-5613), or ziftomenib (KO-539) alongside the BCL2 inhibitor venetoclax, FLT3 inhibitors, or immunoproteasome-targeting agents for *KMT2A*-rearranged and *NPM1*-mutated leukemia, regardless of the *FLT3* and *TP53* mutation status [27,28,29,30,31]. To date, revumenib stands as the most clinically developed small molecule menin inhibitor, demonstrating promising results in the first in-human phase I/II clinical trials including relapsed or refractory pediatric and adult *KMT2A*-rearranged or *NPM1*-mutated acute leukemia patients [32]. However, the number of ALL patients treated in these studies remains limited, with pediatric ALL patient data being particularly scarce [33]. Moreover, despite an impressive 33% of *KMT2A*-rearranged leukemia patients achieving complete remission (CR), progressive disease and relapse occur due to innate or acquired resistance. This may be attributed to the acquisition of mutations in *MEN1* (the gene encoding menin) preventing correct binding of the inhibitor [34]. Collectively, these findings underscore the necessity for additional preclinical and meticulous clinical investigations to determine which patients might actually benefit from this type of targeted therapy.

In the present study, we aimed to delve deeper into the potential use of revumenib for the treatment of high-risk *KMT2A*-rearranged ALL, as so far most preclinical studies focused on *KMT2A*-rearranged AML. We uncovered profound differences in the (type of) responses to revumenib between *KMT2A*-rearranged ALL and AML. In addition, we show the tremendous synergy between the menin inhibitor revumenib and the DOT1L inhibitor pinometostat specifically in *KMT2A*-rearranged ALL.

## 2. Results

### 2.1. Diverse Responses to Revumenib in KMT2A-Rearranged ALL and AML Samples

**Figure 1:**
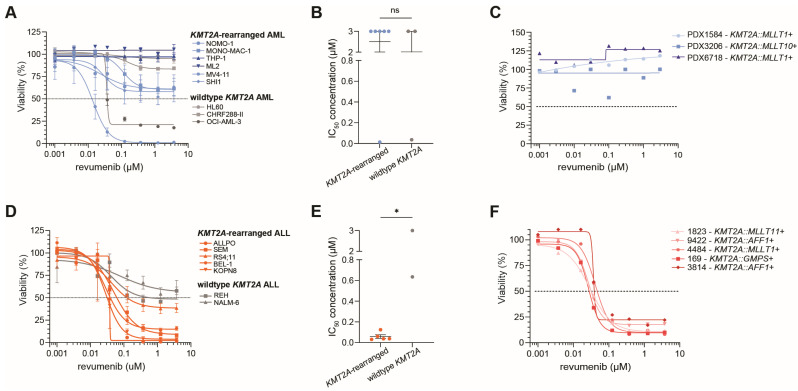
Responses to revumenib in *KMT2A*-rearranged AML and ALL cells. (**A**) Cell viability in response to increasing concentrations of revumenib as assessed by 4-day MTT assays in *KMT2A*-rearranged (n = 6) and wildtype *KMT2A* (n = 3) AML cell line models. The dashed line shows the 50% viability threshold. Experiments were performed in technical triplicates and data consisted of three biological replicates. (**B**) IC_50_-values (i.e., the inhibitory concentration to 50% of the leukemic cells) for revumenib as determined by nonlinear regression in *KMT2A*-rearranged and wildtype *KMT2A* AML cell lines, statistically evaluated by an unpaired two-tailed *t*-test, with ns showing no significant differences. (**C**) Cell viability in response to increasing concentrations of revumenib using 4-day MTT assays in ex vivo pediatric *KMT2A*-rearranged AML patient samples obtained from patient-derived xenograft mouse models (n = 3). The dashed line shows the 50% viability threshold. Experiments were performed in technical triplicates. (**D**) Cell viability in response to increasing concentrations of revumenib as assessed by 4-day MTT assays in *KMT2A*-rearranged ALL cell line models (n = 5) and wildtype *KMT2A* ALL cell lines (n = 2). The dashed line shows the 50% viability threshold. Experiments were performed in technical triplicates and data consisted of three biological replicates. (**E**) IC_50_-values for revumenib as determined by nonlinear regression in *KMT2A*-rearranged and wildtype *KMT2A* ALL cell lines, statistically evaluated by an unpaired two-tailed *t*-test; * *p* < 0.05 (**F**) Cell viability in response to increasing concentrations of revumenib using 4-day MTT assays in ex vivo pediatric *KMT2A*-rearranged ALL patient samples (n = 5). The dashed line shows the 50% viability threshold Experiments were performed in technical triplicates.

To verify the specificity of revumenib for *KMT2A*-rearranged acute leukemias, 4-day MTT assays were performed on *KMT2A*-rearranged as well as wildtype *KMT2A* ALL and AML cell lines. The *KMT2A*-rearranged AML cell lines did not demonstrate sensitivity to revumenib as compared with the wildtype *KMT2A* AML cell line model. In hardly any of the cell lines, an IC_50_-value (i.e., the drug concentration inhibitory to 50% of the leukemic cells) was reached even at the highest tested concentration of 3 µM of revumenib, with the exception of MV4-11 and OCI-AML-3 (Figure 1A,B). Given that plasma levels of revumenib in leukemia patients seldom exceed 3 µM [32], this concentration was chosen as the upper limit for our study to reflect clinically relevant dosing. MV4-11 represents a *KMT2A*-rearranged AML cell line carrying a *KMT2A* fusion that is typically associated with ALL and that is only sporadically detected in AML patients, and OCI-AML-3 is an *NPM1*-mutated AML cell line known to be sensitive to revumenib due to its exceptional dependency on menin [35]. Results from 4-day MTT assays performed on ex vivo PDX-derived pediatric *KMT2A*-rearranged AML patient samples confirmed a complete lack of response to revumenib at all tested concentrations (Figure 1C).

In contrast, similar experiments in ALL cell lines showed significant differences in their response to revumenib between *KMT2A*-rearranged and wild-type *KMT2A* ALL cell line models (Figure 1D,E). All *KMT2A*-rearranged ALL cell lines were highly sensitive to revumenib with IC_50_-values ranging between 0.031 µM and 0.125 µM compared to IC_50_-values of 0.635 µM and >3 µM in wildtype *KMT2A* ALL lines (Figure 1E). Importantly, results from 4-day viability assays on primary *KMT2A*-rearranged infant ALL patient samples confirmed tremendous sensitivity to revumenib with IC_50_-values of <0.05 µM in all samples irrespective of the *KMT2A* fusion partner (Figure 1F).

Taken together, these data reveal a notable difference in the in vitro response to revumenib between *KMT2A*-rearranged ALL and *KMT2A*-rearranged AML as measured by 4-day MTT assays (Supplementary Figure S1).

### 2.2. Delayed Revumenib Responses and Differentiation in KMT2A-Rearranged AML Cells

**Figure 2:**
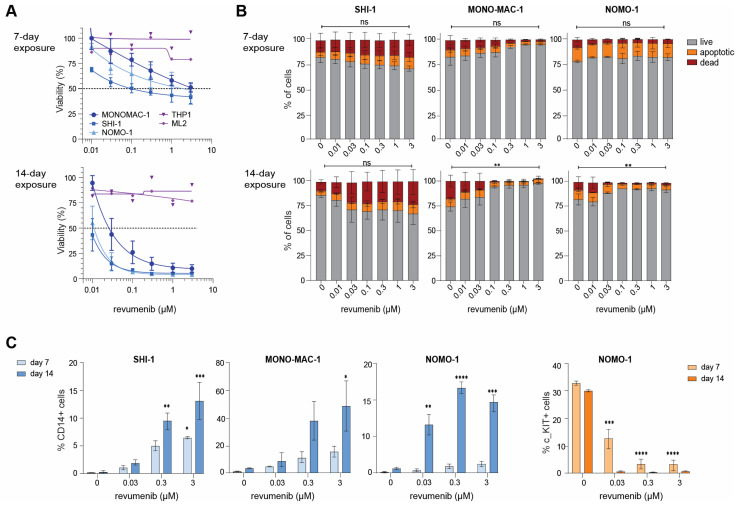
Prolonged revumenib exposure induces myeloid differentiation in *KMT2A*-rearranged AML cells. (**A**) Percentage of viable cells after 7 and 14-day exposures to indicated concentrations of revumenib as determined by trypan blue exclusion in *KMT2A*-rearranged AML cell line models (n = 5). Responsive cell lines are in blue and unresponsive cell lines are in purple. The dashed line shows the 50% viability threshold. Experiments were performed in technical duplicates and data consisted of two biological replicates. (**B**) Percentages of live (grey), apoptotic (orange), and dead (red) cells after 7 and 14-day exposures to indicated concentrations of revumenib as determined by an Annexin V/7AAD staining and flowcytometry in *KMT2A*-rearranged AML cell line models. Differences in live, apoptotic, and death cells induced by revumenib as compared to untreated controls were statistically verified by two-way ANOVA Tukey’s multiple comparisons tests. Data consisted of two biological replicates. (**C**) Flow cytometric assessment of the expression of the myeloid differentiation marker CD14 and of stem cell marker CD117 (c-Kit) after 7 and 14 days of revumenib exposure in the responsive *KMT2A*-rearranged AML cell lines SHI-1, MONO-MAC-1, and NOMO-1, from duplicate experiments. * *p* < 0.05, ** *p* < 0.005, *** *p* < 0.0005, **** *p* < 0.00005 and ns for no significant *p*.

Attempting to invoke a response, we exposed *KMT2A*-rearranged AML cell lines for 7 and 14 days to various concentrations of revumenib and assessed cell viability by Trypan blue exclusion tests. Interestingly, this led to responses in three of the five cell lines tested, i.e., NOMO-1, MONO-MAC-1, and SHI-1, while the cell line models THP1 and ML2 remained non-responsive even after 14 days (Figure 2A). As resistance to menin inhibition may involve mutations in *MEN1* [34], we sequenced the entire *MEN1* gene in all *KMT2A*-rearranged AML cell lines and found no mutations that could explain the delayed or complete lack of responses in these cell lines (Supplementary Table S1).

To assess the cause of the reduction in viable cells in response to revumenib exposure over time in the *KMT2A*-rearranged AML cell lines NOMO-1, MONO-MAC-1, and SHI-1 (Figure 2A), we conducted apoptosis, cell cycle, and myeloid differentiation CD14/CD117 FACS assays on day 7 and day 14. In SHI-1 cells, we observed a very modest and dose-dependent increase in apoptotic and dead cells on Day 7 and Day 14 whereas in MONO-MAC-1 and NOMO-1 the percentage of live cells was found to be slightly increased in response to revumenib exposure (Figure 2B, Supplementary Table S2). Hence, *KMT2A*-rearranged AML cell lines displaying delayed responses to revumenib are not subjected to substantial apoptosis induction. Subsequent cell cycle analysis revealed no effects in SHI-1 whereas small decreases in the S-phase and increases in the G1-phase were observed in MONO-MAC-1 and NOMO-1, suggesting that subsets of these cells become arrested in the G1-phase (Supplementary Figure S2A and Table S3). Finally, myeloid differentiation in response to revumenib was assessed by flow cytometric analysis of monocyte and macrophage differentiation marker CD14. This revealed a significant increase in CD14^+^ cells in all three cell lines over time (Figure 2C, Supplementary Table S3), indicating that revumenib is markedly inducing myeloid differentiation in these cells. In line with this, we found the one cell line (i.e., NOMO-1) expressing the stem cell marker CD117 (c-Kit) to completely lose CD117 expression over time (Figure 2C, Supplementary Table S4).

Thus, in *KMT2A*-rearranged AML cell populations showing slow responses to prolonged exposure to revumenib, reductions in viable cells are largely caused by cell cycle arrest allowing subsequent myeloid differentiation, occasionally accompanied by a modest induction of apoptosis.

### 2.3. Rapid Apoptosis Induction in Response to Revumenib in KMT2A-Rearranged ALL Cells

**Figure 3:**
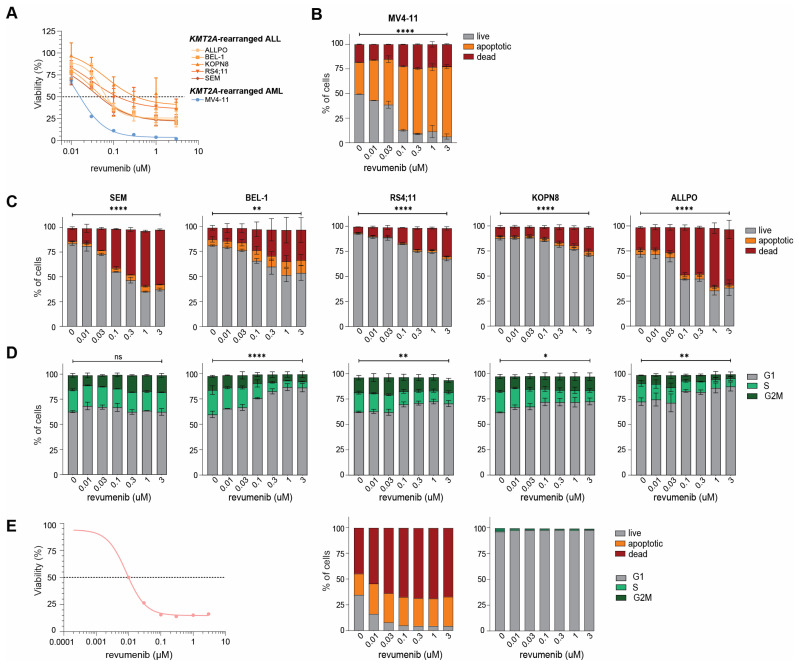
Revumenib readily induces apoptosis in *KMT2A*-rearranged ALL cells. (**A**) Percentage of viable cells after 4-day exposures to indicated concentrations of revumenib as determined by trypan blue exclusion in *KMT2A*-rearranged ALL cell line models (n = 5; in orange) and the highly sensitive *KMT2A*-rearranged AML cell line MV4-11 (in blue). The dashed line shows the 50% viability threshold. Experiments were performed in technical duplicates and data consisted of two biological replicates. (**B**) Percentages of live (grey), apoptotic (orange), and dead (red) cells after 4-day exposures to indicated concentrations of revumenib as determined by flow cytometry and Annexin V/7AAD staining in the *KMT2A*-rearranged AML cell line MV4-11 and (**C**) in the *KMT2A*-rearranged ALL cell lines. Differences in live, apoptotic, and death cells induced by revumenib as compared to untreated controls were statistically verified by two-way ANOVA Tukey’s multiple comparisons tests. Data consisted of two biological replicates. (**D**) Cell cycle analysis showing the percentages of cells residing in the G1-phase, S-phase, and GM2-phase as determined by Hoechst 33342/7AAD staining and flow cytometry after 4-day exposures to indicated concentrations of revumenib. Differences in cell cycle phases induced by revumenib as compared to untreated controls were statistically verified by two-way ANOVA Tukey’s multiple comparisons tests. Data consisted of two biological replicates. (**E**) Cell viability (as determined by trypan blue exclusion) and cell cycle analysis (as determined by Hoechst 33342/7AAD staining and flow cytometry) after 4-day exposures to indicated concentrations of revumenib in a representative *KMT2A*-rearranged infant ALL patient sample obtained from a patient-derived xenograft mouse model. The dashed line shows the 50% viability threshold. * *p* < 0.05, ** *p* < 0.005, **** *p* < 0.00005 and ns for no significant *p*.

To validate our earlier metabolic-based 4-day MTT-assay results (Figure 1D,E), we performed flow cytometry-based 7-AAD cell viability assays on *KMT2A*-rearranged ALL cell lines exposed to varying concentrations of revumenib. Given its remarkable and fast response to revumenib in vitro, we also included the *KMT2A*-rearranged AML cell line MV4-11 in these experiments. The results from these assays confirmed that these cell lines are indeed highly sensitive to revumenib with IC_50_-values ranging between 0.0455 µM and 0.341 µM (Figure 3A).

Subsequent apoptosis assays revealed a significant and dose-dependent increase in apoptotic and dead cells in the cell lines MV4-11, SEM, BEL-1, RS4;11, KOPN8, and ALLPO (Figure 3B,C; Supplementary Tables S5 and S7). Cell cycle analysis showed that in most of the *KMT2A*-rearranged ALL cell lines, increased numbers of cells became arrested in the G1-phase upon revumenib exposure, with the exception of SEM cells (Figure 3D, Supplementary Table S8). Considering the tremendous amount of apoptosis induction and cell death in MV4-11 (Figure 3B), we deemed the cell cycle data obtained from viable cells in this cell line as unreliable (Supplementary Figure S2B and Table S6). Similar experiments in a primary *KMT2A*-rearranged infant ALL patient sample confirmed the induction of apoptosis and increased numbers of dead cells by revumenib (Figure 3E).

### 2.4. Acquired Resistance to Revumenib in KMT2A-Rearranged ALL Cells

**Figure 4:**
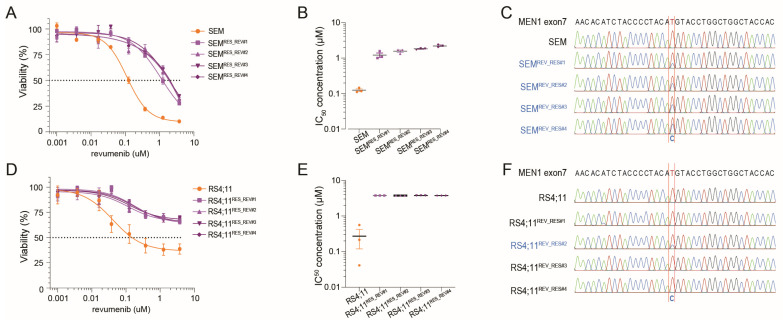
Induction of acquired resistance to revumenib in *KMT2A*-rearranged ALL cells. Induction of acquired resistance to revumenib was accomplished by exposing the *KMT2A::AFF1*^+^ ALL cell lines SEM and RS4;11 to increasing concentrations of revumenib of up to 10 µM for 10 weeks. Revumenib-resistant daughter cell lines are indicated as SEM^REV_RES#1–4^ and RS4;11^REV_RES#1–4^, respectively. (**A**) Cell viability in response to increasing concentrations of revumenib as assessed by 4-day MTT assays in SEM (orange) and revumenib-resistant daughter cell lines SEM^REV_RES#1–4^(purple). The dashed line shows the 50% viability threshold. Experiments were performed in technical triplicates and data consisted of three biological replicates. (**B**) IC_50_-values for revumenib as determined by nonlinear regression in SEM (orange) and revumenib-resistant daughter cell lines SEM^REV_RES#1–4^ (purple) evaluated by an unpaired two-tailed *t*-test. (**C**) *MEN1* mutation analysis (i.e., Sanger sequencing results) showing an M322T *MEN1* mutation in all revumenib-resistant SEM daughter lines SEM^REV_RES#1–4^ (blue). (**D**) Cell viability in response to increasing concentrations of revumenib as assessed by 4-day MTT assays in RS4;11 (orange) and revumenib-resistant daughter cell lines RS4;11^REV_RES#1–4^ (purple). The dashed line shows the 50% viability threshold. Experiments were performed in technical triplicates and data consisted of three biological replicates. (**E**) IC_50_-values for revumenib as determined by nonlinear regression in RS4;11 (orange) and revumenib-resistant daughter cell lines RS4;11^REV_RES#1–4^ (purple) evaluated by an unpaired two-tailed *t*-test. (**F**) *MEN1* mutation analysis reveals an M322T *MEN1* mutation only in revumenib-resistant RS4;11^REV_RES#2^ cells (blue).

Acquired resistance to menin inhibition as a result of gaining mutations in the *MEN1* gene is frequently observed in AML patients treated with menin inhibitors [32]. Therefore, we asked whether this would also be true for *KMT2A*-rearranged ALL cells in which menin inhibition rapidly induces apoptosis. We subjected the *KMT2A::AFF1*^+^ ALL cell line SEM to increasing concentrations of revumenib to a maximum of 10 µM over a period of 10 weeks. Remarkably, after 10 weeks of sustained revumenib exposure, various daughter lines of SEM designated here as SEM^REV_RES#1^, SEM^REV_RES#2^, SEM^REV_RES#3^, and SEM^REV_RES#4^ readily acquired resistance to menin inhibition (Figure 4A). Compared to naïve SEM cells, the revumenib-resistant daughter cell lines showed a 10 to 20-fold increase in IC_50_-values (Figure 4B). All resistant daughter lines acquired an M322T mutation in exon 7 of *MEN1* (encoding the revumenib binding pocket in menin), which is absent in the original SEM cell line (Figure 4C). Intriguingly, inducing revumenib resistance in the *KMT2A::AFF1*^+^ ALL cell line RS4;11 cell line (Figure 4D,E), led to the acquisition of the *MEN1* M322T mutation in only one (i.e., RS4;11^REV_RES#2^) of four revumenib-resistant daughter lines of RS4;11. In the remaining three daughter cell lines no mutations were detected after sequencing the entire *MEN1* gene. Hence, like in AML, acquired resistance to menin inhibition in *KMT2A*-rearranged ALL does not necessarily require the acquisition of *MEN1* mutations.

### 2.5. Synergy between Revumenib and Pinometostat in KMT2A-Rearranged ALL

**Figure 5:**
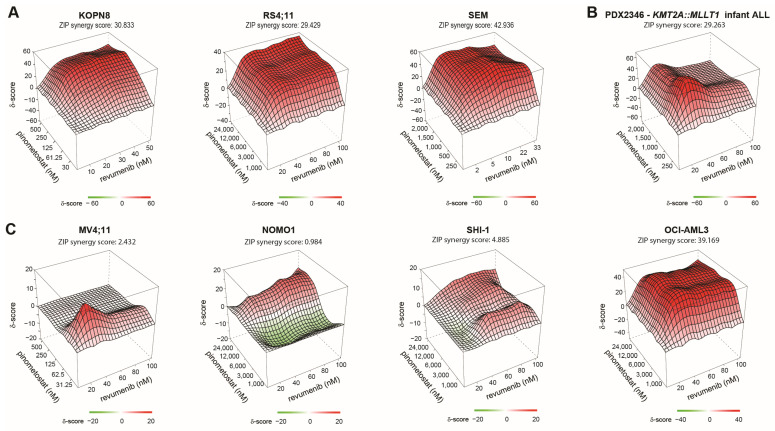
Synergy between revumenib and pinometostat in *KMT2A*-rearranged ALL. Three-dimensional synergy plots showing drug synergy (red) or antagonism (green) between indicated concentrations of revumenib (x-axis) and pinometostat (y-axis) as determined by 6-day pinometostat pre-treated cells followed by 4-day MTT assays of revumenib exposures in (**A**) *KMT2A*-rearranged ALL cell lines, (**B**) a PDX derived *KMT2A*-rearranged infant ALL patient sample, and (**C**) *KMT2A*-rearranged AML cell lines. Drug synergy/antagonism is expressed as Zero Interaction Potency (ZIP) scores (z-axis), with scores of >5 being considered as synergistic effects (red areas), and ZIP scores below −5 are deemed as antagonistic effects (green areas). On top of each 3D synergy plot, the average ZIP score over the entire range of pinometostat and revumenib concentrations is listed.

Recent data on the predecessor of revumenib, i.e., SDX-50469, revealed that both menin and DOT1L inhibition lead to similar disruptions of KMT2A-fusion-driven gene expression programs [25]. Therefore, we opted here to investigate the potential synergy between revumenib and the DOT1L inhibitor pinometostat (EPZ-5676) in *KMT2A*-rearranged acute leukemic cells. For this, leukemic cells were pre-treated with pinometostat for 6 days prior to 4-day MTT assays combining revumenib and pinometostat [32,36]. In *KMT2A*-rearranged ALL cell lines, combined exposure to low nM concentrations of each drug revealed high levels of synergistic lethality, with synergy scores (i.e., Zero Interaction Potency (ZIP) scores) ranging between 29.6 and 42.9 (while ZIP scores > 5 are already considered to represent a synergistic effect) (Figure 5A). Verification of the synergy findings in a patient setting was performed using PDX material from a *KMT2A*-rearranged infant ALL patient sample, selected for its ability to be cultured in vitro for longer periods, thereby confirming the observed synergy in cell lines. (Figure 5B). The corresponding dose-response matrixes demonstrate synergistic inhibition at all concentrations in any combination of revumenib and pinometostat (Supplementary Figure S3A,B). In contrast, in the revumenib-responsive *KMT2A*-rearranged AML cell lines NOMO-1 and SHI-1 (Figure 2A), the beneficial effects of combined exposure to revumenib and pinometostat were minimal with ZIP scores of 0.98 and 4.89, respectively (Figure 5C). Determining synergistic effects in the MV4-11 cell line, which exhibits exceptional responsiveness to revumenib as a *KMT2A*-rearranged AML (Figure 1A), was difficult to discern due to the tremendous amount of induced cell death. Consequently, synergy was only observed specifically at the lowest concentrations (Figure 5C and Supplementary Figure S3C). Finally, the *NPM1*-mutated (wildtype *KMT2A*) AML cell line OCI-AML-3 displayed a marked synergistic effect comparable to that observed in *KMT2A*-rearranged ALL cells (Figure 5C and Supplementary Figure S3B).

## 3. Discussion

Therapeutic disruption of the interactions between menin and the KMT2A-fusion protein complex has demonstrated single-agent clinical responses for patients with *KMT2A*-rearranged acute leukemia [19,20,21,22,23,24,25,26]. To optimize the clinical success of menin inhibitors, it is important to understand which patients are likely to benefit from these agents and which patients will not. Therefore, we here studied the in vitro response to revumenib in both *KMT2A*-rearranged ALL and AML. *KMT2A*-rearranged ALL samples uniformly responded swiftly as a result of substantial and dose-dependent induction of leukemic cell death within 4 days. In *KMT2A*-rearranged AML, revumenib appeared to promote myeloid differentiation rather than apoptosis, as observed in *KMT2A*-rearranged ALL. One exception, however, was the *KMT2A*-rearranged AML cell line MV4-11, which was highly sensitive to revumenib due to severe induction of apoptosis in a short period of time. MV4-11 carries the t(4;11) translocation giving rise to the *KMT2A*::*AFF1* fusion gene which represents the most common *KMT2A* fusion in ALL but is only rarely observed among AML patients. Therefore, caution is advised when using this cell line as a representation of *KMT2A*-rearranged AML in drug response studies on menin inhibition.

As shown, some of the *KMT2A*-rearranged AML cell lines remained unresponsive to revumenib after 14 days of exposure, suggesting that these models may be more resistant compared to others. Remarkably, the unresponsive ML2 and THP1 did show in vitro responses to SNDX-50469 (VTP50469), the predecessor of revumenib (SNDX-5613), although it is unclear how long these cell lines were exposed [25]. Different menin inhibitors, such as SNDX-50469 or ziftomenib, may yield different outcomes compared to revumenib. Further preclinical studies are needed to explore these differences.

Interestingly, however, these unresponsive AML models do not carry somatic mutations in *MEN1* which often drive acquired resistance to revumenib [34]. On the other hand, mutations in *MEN1* were gained in *KMT2A*-rearranged ALL cells that became resistant to revumenib by prolonged exposure, indicating that the acquisition of *MEN1* mutations plays an important role in acquired revumenib resistance in *KMT2A*-rearranged ALL. Yet, in another *KMT2A*-rearranged ALL cell line, acquired revumenib resistance was developed in the absence of *MEN1* mutations, suggesting alternative mechanisms of reduced responsiveness to menin inhibition. Hence, simply monitoring the *MEN1* mutation status alone before and/or during treatment to identify potential non-responding patients may well be inadequate. Discovering alternative resistance mechanisms not relying on *MEN1* mutations and identifying potential biomarkers that predict responsiveness to revumenib treatment is imperative.

Like targeting menin, the inhibition of DOT1L using small molecule inhibitors represents a promising avenue for targeting the KMT2A-fusion protein complex but is even more susceptive to the induction of acquired resistance upon prolonged exposure in vitro and in clinical trial(s) [36,37,38]. Possibly, combinatorial treatment of various therapeutic agents disrupting the same oncogenic multiprotein complex is potentially more efficient and may avoid the development of acquired resistance [39]. Previous studies showed synergistic effects by combining the predecessor of revumenib, SNDX-50469 (VTP50469), with a DOT1L inhibitor in both *KMT2A*-rearranged and *NPM1*-mutated acute leukemia [35,40]. We validated this by demonstrating pronounced synergy between the menin inhibitor revumenib and the DOT1L inhibitor pinometostat, especially in *KMT2A*-rearranged ALL cells. Hence, simultaneously targeting menin and DOT1L may represent a therapeutic strategy worth exploring in *KMT2A*-rearranged ALL patients, especially since improved inhibitors with more favorable pharmacokinetic profiles are being developed [41,42,43]. The combined inhibition of menin and DOT1L may even be complemented by additional agents specifically disrupting critical interactions in KMT2A-fusion protein complexes, such as, for instance, BRD4 inhibitors [44,45]. Additionally, combining these therapies with blinatumomab, a bispecific T-cell engager molecule targeting CD19, which has recently shown significant efficacy in infants diagnosed with KMT2A-rearranged ALL, could potentially enhance their therapeutic effects [7].

## 4. Materials and Methods

### 4.1. Patient Samples

Primary *KMT2A*-rearranged infant ALL patient specimens were collected as part of the INTERFANT studies [6,46]. Informed consent was obtained from parents or legal guardians to use excess diagnostic material for research purposes, as approved by the institutional review board. These studies were conducted in accordance with the Declaration of Helsinki. All samples were processed and cultured as previously described [47]. The leukemic bone marrow aspirates and blood samples were obtained at diagnosis and contained >90% blasts, as determined by May–Grünwald Giemsa (Merck, Rahway, NJ, USA) stained cytospins.

### 4.2. Cell Line Models

The *KMT2A*-rearranged B-ALL cell lines utilized in this study include SEM (*KMT2A::AFF1*^+^; DSMZ (German collection of microorganisms and cell cultures GmbH, Göttingen, Germany): ACC 546), KOPN-8 (*KMT2A::MLLT1*^+^; DSMZ: ACC 552), RS4;11 (*KMT2A::AFF1*^+^; ATCC (American Type Culture Collection, Mannassas, VA, USA): CRL-1873), BEL-1 (*KMT2A::AFF1*^+^) and ALL-PO (*KMT2A::AFF1*^+^). BEL-1 was a kind gift from the lab of Dr. Ruoping Tang (University Laboratory, Paris, France) and ALL-PO from the lab of Prof. dr. Cazzaniga (University of Milano-Bicocca, Monza, Italy) [48]. The wildtype *KMT2A* B-cell precursor (BCP) ALL cell lines include REH (carrying translocation t(12;21); DMSZ: ACC 22) and NALM-6 (carrying translocation t(5;12); DSMZ: ACC 128). The *KMT2A*-rearranged AML cell lines include MV4-11 (*KMT2A::AFF1*^+^; DMSZ: ACC 102), ML2 (*KMT2A::AFDN*^+^; DMSZ: ACC 15), SHI-1 (*KMT2A::AFDN*^+^; DSMZ: ACC 645), NOMO-1 (*KMT2A::MLLT3*^+^; DSMZ: ACC 542), THP-1 (*KMT2A::MLLT3*^+^; DSMZ: ACC 16), and MONO-MAC-1 (*KMT2A::MLLT3*^+^; DSMZ: ACC 252), and the wildtype *KMT2A* AML cell lines include HL-60 (DMSZ: ACC 3), Kasumi-1 (AML1-ETO); DSMZ: ACC 220), and OCI-AML3 (NPM1 mutated; DSMZ: ACC 582). All leukemia cell lines were cultured in RPMI-1640 medium containing GlutaMAX^TM^ supplemented with 10–20% fetal calf serum, 100 IU/mL Penicillin and Streptomycin (Thermo Fisher Scientific, Waltham, MA, USA), and 0.125 µg/mL Amphotericin B (Life Technologies, Carlsbad, CA, USA), at 37 °C under a 5% CO_2_-containing atmosphere. All cell lines were routinely tested for the absence of mycoplasma and were DNA fingerprinted to assure cell line authenticity.

### 4.3. Therapeutic Agents and Viability Assays

Revumenib (SNDX-5613: Cat. no. HY-136175) was purchased from MedChemExpress (Princeton, NJ, USA) and pinometostat (EPZ5676: Cat. no. S7062) was purchased from Selleckchem (Houston, TX, USA). Both compounds were dissolved in DMSO and stored at −20 °C in small aliquots. Compounds were thawed and directly diluted into a warm culture medium when used for drug exposure experiments and cell viability assays.

Cells were seeded into 384-well plates (Corning, New York, NY, USA) using the Multidrop (Thermo Fisher Scientific) and drugs were added by a Hewlett-Packard D300 Digital Dispenser (Tecan, Männedorf, Switzerland). Cell viability was assessed by 4-day thiazolyl blue tetrazolium bromide (MTT) assays as described elsewhere [49]. In short, after 4 days of incubation of cells with revumenib and/or pinometostat, 5 µL/well MTT (5 mg/mL, Sigma-Aldrich, Saint Louis, MO, USA) was added to the 40 µL cell suspension and incubated for 5 h. Next, the reaction was stopped using 40 µL of a 10% SDS/0.01 M HCL solution per well and after 24 h the absorbance was measured at wavelengths of 570 nm and 720 nm using the SpectraMax iD3 microplatereader (Molecular Devices, San Jose, CA, USA). MTT data were normalized to DMSO controls and the maximum concentration of DMSO was 0.5% to minimize toxicity induced by the solvent.

### 4.4. In Vitro Exposure to Revumenib and Trypan Blue Exclusion

Leukemia cells were seeded in T25 flasks (Corning, New York, NY, USA) at an optimal density for growth and increasing amounts of revumenib (0 µM; 0.01 µM; 0.01 µM; 0.03 µM; 0.1 µM; 0.3 µM; 1 µM; and 3 µM) were added to the culture media. Cells were counted manually using Trypan blue exclusion to distinguish between viable (white) and dead (blue) cells and passaged each 3–4 days into fresh culture media with newly added concentrations of revumenib. At all passages, cells were collected for flow cytometric determination of cell viability, cell cycle, apoptosis assay, and/or CD marker expression analysis.

### 4.5. Establishment of Acquired Revumenib Resistance in SEM Cells

SEM cells were cultured in the presence of gradually increasing revumenib concentrations (ranging from 2 nM up to 2.5 µM) for a period of 10 weeks. For assessment of the in vitro response to revumenib, cells were cultured in the absence of revumenib for several passages before determining cell viability in response to revumenib exposure using 4-day MTT assays (see above).

### 4.6. Flow Cytometry (FACS) Analyses

All FACS analysis experiments were performed on a CytoFlex S Flow Cytometer (Beckman Coulter, Woerden, The Netherlands). To determine cell viability, an apoptosis assay was performed by staining the collected leukemic cells with Annexin V-FITC (Biolegend, San Diego, CA, USA) and 7AAD (Biolegend) in Annexin V buffer following the manufacturer’s protocol. Additionally, cell cycle analysis was performed by incubating live cells with Hoechst 33342 (1 µg/mL, Thermo Fisher Scientific) for 1 h. Next, 7AAD staining was added to the suspension for subsequent dead cell discrimination.

To determine CD marker protein expression on AML cells, cells were labeled with eBioscience^TM^ Fixable Viability Dye eFluor^TM^ 450 (Invitrogen, Waltham, MA, USA) to select for viable cells, blocked with Human TruStain FcX™ (BioLegend), and stained with CD38-FITC (Biolegend, #980304), CD117(c-Kit)-APC (Biolegend, #313206) and CD14-PE (BD Pharmingen^TM^, San Diego, CA, USA, #555398) according to the manufacturer’s recommendation. Raw CytoFLEX S data were processed using FlowJo^TM^ software version 10.9.0 (BD Biosciences, San Diego, CA, USA).

### 4.7. Drug Synergy Experiments

Cell lines were pre-treated with various pinometostat concentrations or an equivalent volume of DMSO (vehicle controls, maximum concentration of 0.5% *v*/*v*) for six days, with passaging on day four. Subsequently, cells were seeded into 384-well plates at 10,000 cells per well, treated with specified pinometostat concentrations, and exposed to various revumenib concentrations using a Hewlett-Packard D300 Digital Dispenser (Tecan). Cell viability was assessed using 4-day MTT assays, as described in Section 4.3 ‘Therapeutic Agents and Viability Assays’. Viability measurements were normalized to the DMSO controls. Drug synergy analysis was conducted using the SynergyFinder web application https://synergyfinder.fimm.fi/, accessed between 18 April 2023 and 1 May 2024 [50]. This online tool facilitates the assessment of drug interactions within combination therapy regimens. Zero Interaction Potency (ZIP) scores, a quantitative measure of drug synergy, were employed to evaluate the degree of interaction between the administered drugs. Specifically, the ZIP score is calculated as the logarithm of the observed effect divided by the expected effect under the assumption of no interaction (additivity). A ZIP score exceeding 5 is conventionally considered indicative of synergistic drug interactions, suggesting a cumulative effect greater than the sum of individual drug effects.

### 4.8. Menin Mutation Analysis

For mutation analysis, genomic DNA from all cell lines was isolated using the QIAamp DNA Micro Kit (Qiagen, Hilden, Germany), following the manufacturer’s protocol. Mutation detection was carried out through PCR amplification and Sanger sequencing of the entire *MEN1* gene, followed by analysis using the CLC Workbench software version 3.0 (CLC Bio, a Qiagen Company). The primer sequences employed are provided in Supplementary Table S9.

### 4.9. Statistical Analysis

GraphPad Prism version 10.0.2 software was used for statistical analysis and data are represented as means ± SEM. Statistical significance was determined by *t*-test (two-tailed). Differences between groups were considered statistically significant with a *p*-value cutoff of 0.05 and is represented as *; *p* < 0.01 as **; *p* < 0.001 as ***; *p* < 0.0001 as ****; and not significant as n.s.

## Data Availability

Any information required to reanalyze the data reported in this paper is available from the lead contact upon request.

## Supplementary Materials

The following supporting information can be downloaded at https://www.mdpi.com/article/10.3390/ijms25116020/s1.
