# Supplementary material for: Distinct Responses to Menin Inhibition and Synergy with DOT1L Inhibition in KMT2A-Rearranged Acute Lymphoblastic and Myeloid Leukemia
Source: Int J Mol Sci. 2024 May 30;25(11):6020. doi: 10.3390/ijms25116020 (PMC11173273; doi:10.3390/ijms25116020)
Supplement: Supplementary file 1 [file ijms-25-06020-s001.zip › supl figure 1_KMT2Ar_Revumenib.pdf]

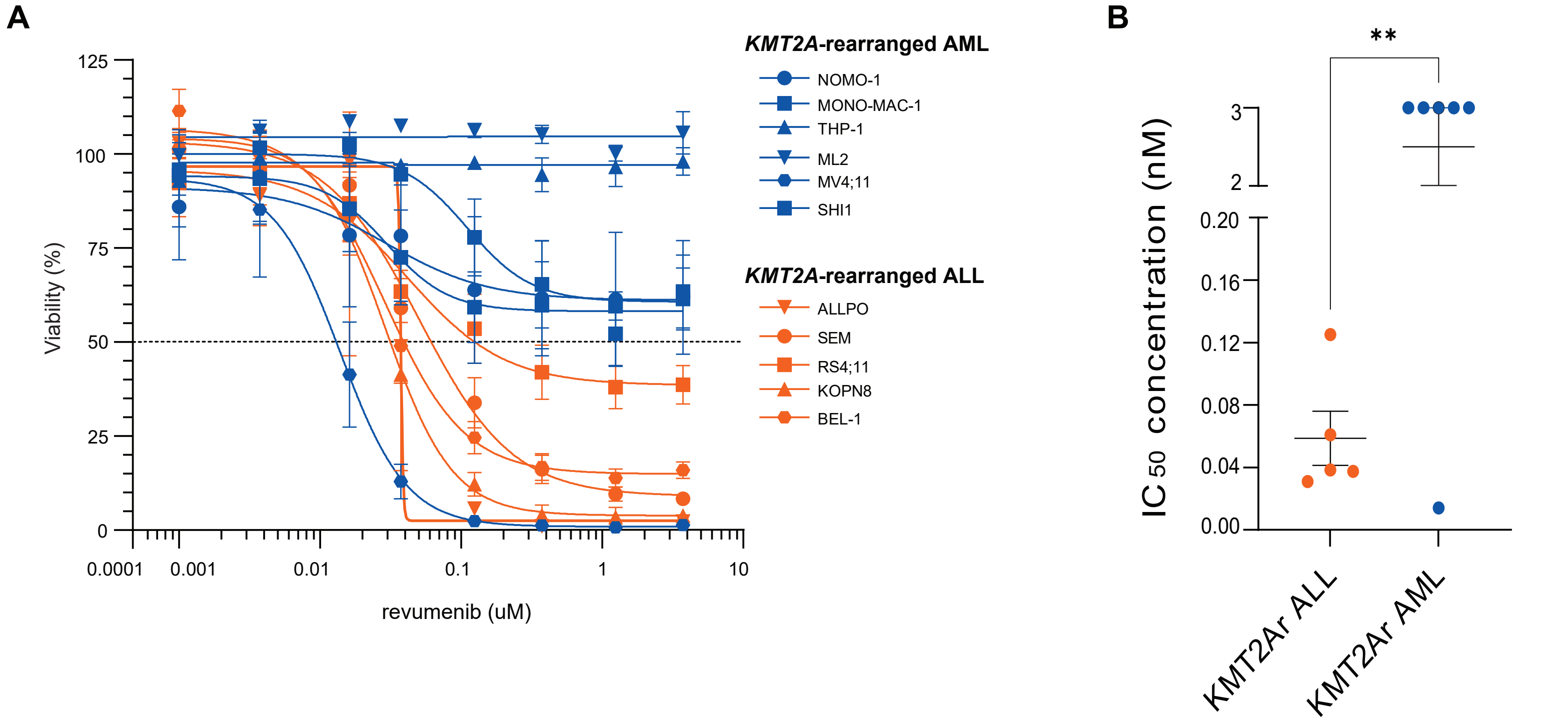

Supplementary Figure S1. Contrasting responses to revumenib between *KMT2A*-rearranged AML and ALL.
(**A)** Cell viability in response to increasing concentrations of revumenib as assessed by 4-day MTT assays in *KMT2A*-rearranged AML (n=6) and *KMT2A*-rearranged ALL (n=5) cell line models. Experiments were performed in technical triplicates and data consists of three biological replicates. (**B**) IC50-values for revumenib as determined by nonlinear regression in *KMT2A*-rearranged ALL and *KMT2A*-rearranged AML cell lines, statistically evaluated by an unpaired 2-tailed t-test.
