# Supplementary material for: Distinct Responses to Menin Inhibition and Synergy with DOT1L Inhibition in KMT2A-Rearranged Acute Lymphoblastic and Myeloid Leukemia
Source: Int J Mol Sci. 2024 May 30;25(11):6020. doi: 10.3390/ijms25116020 (PMC11173273; doi:10.3390/ijms25116020)
Supplement: Supplementary file 1 [file ijms-25-06020-s001.zip › supl figure 2_KMT2Ar_Revumenib.pdf]

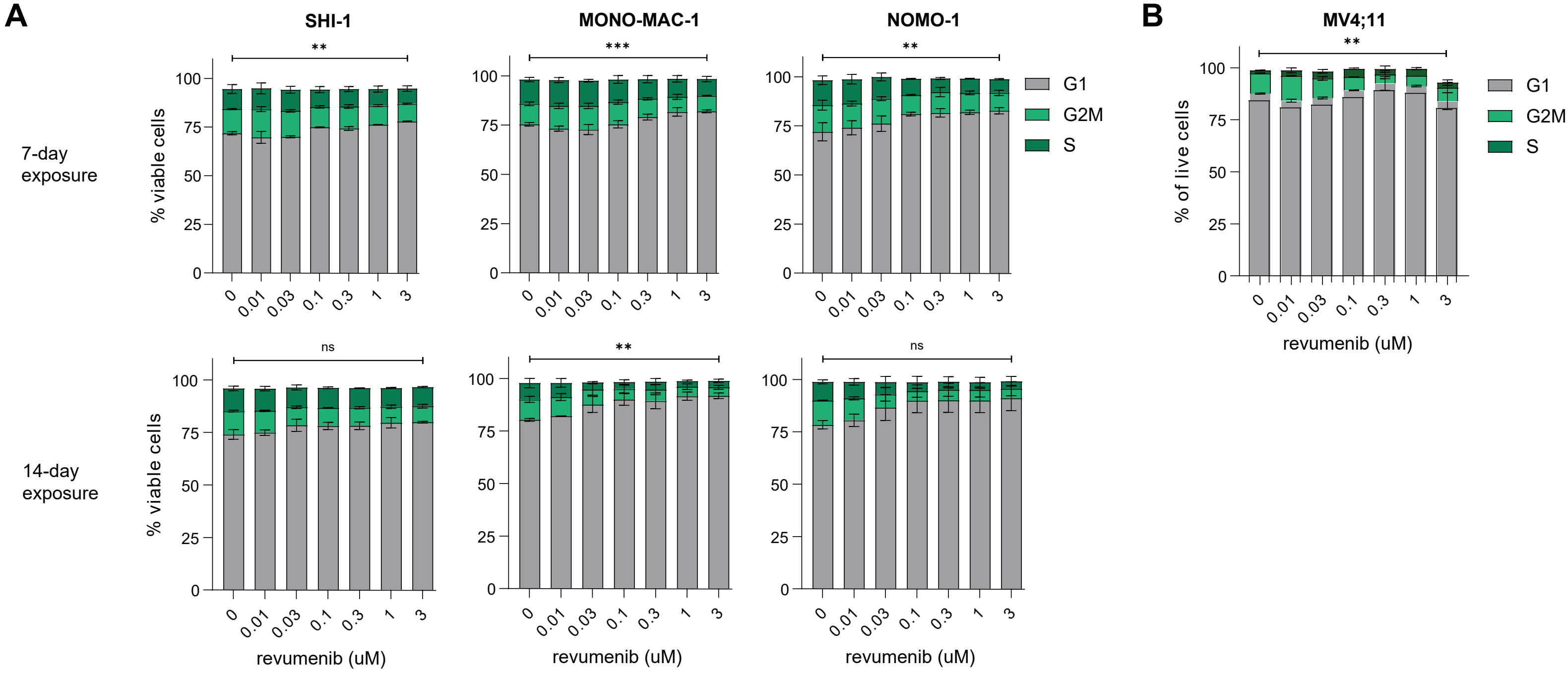

Supplementary Figure S2. Cell cycle analysis in *KMT2A*-rearranged AML cell lines exposed to revumenib.
(**A**) Cell cycle analysis in *KMT2A*-rearranged AML cell line models showing the per-centages of cells residing in the G1-phase, S-phase, and GM2-phase as determined by Hoechst 33342/7AAD staining and flow cytometry after 7 and 14-day exposures to indicated concentrations of revumenib. Differences in cell cycle phases induced by revumenib as compared to untreated controls were statistically verified by 2-way ANOVA Tukey's multiple comparisons tests. Data consists of two biological replicates. (**B**) Cell cycle analysis in *KMT2A*-rearranged AML cell line MV4-11 showing the percentages of cells residing in the G1-phase, S-phase, and GM2-phase as determined by Hoechst 33342/7AAD staining and flow cytometry after 4-day exposures to indi-cated concentrations of revumenib. Differences in cell cycle phases induced by revumenib as compared to untreated controls were statistically verified by 2-way ANOVA Tukey's multiple comparisons tests. Data consists of two biological replicates.
