# Supplementary material for: Distinct Responses to Menin Inhibition and Synergy with DOT1L Inhibition in KMT2A-Rearranged Acute Lymphoblastic and Myeloid Leukemia
Source: Int J Mol Sci. 2024 May 30;25(11):6020. doi: 10.3390/ijms25116020 (PMC11173273; doi:10.3390/ijms25116020)
Supplement: Supplementary file 1 [file ijms-25-06020-s001.zip › supl figure 3_KMT2Ar_Revumenib.pdf]

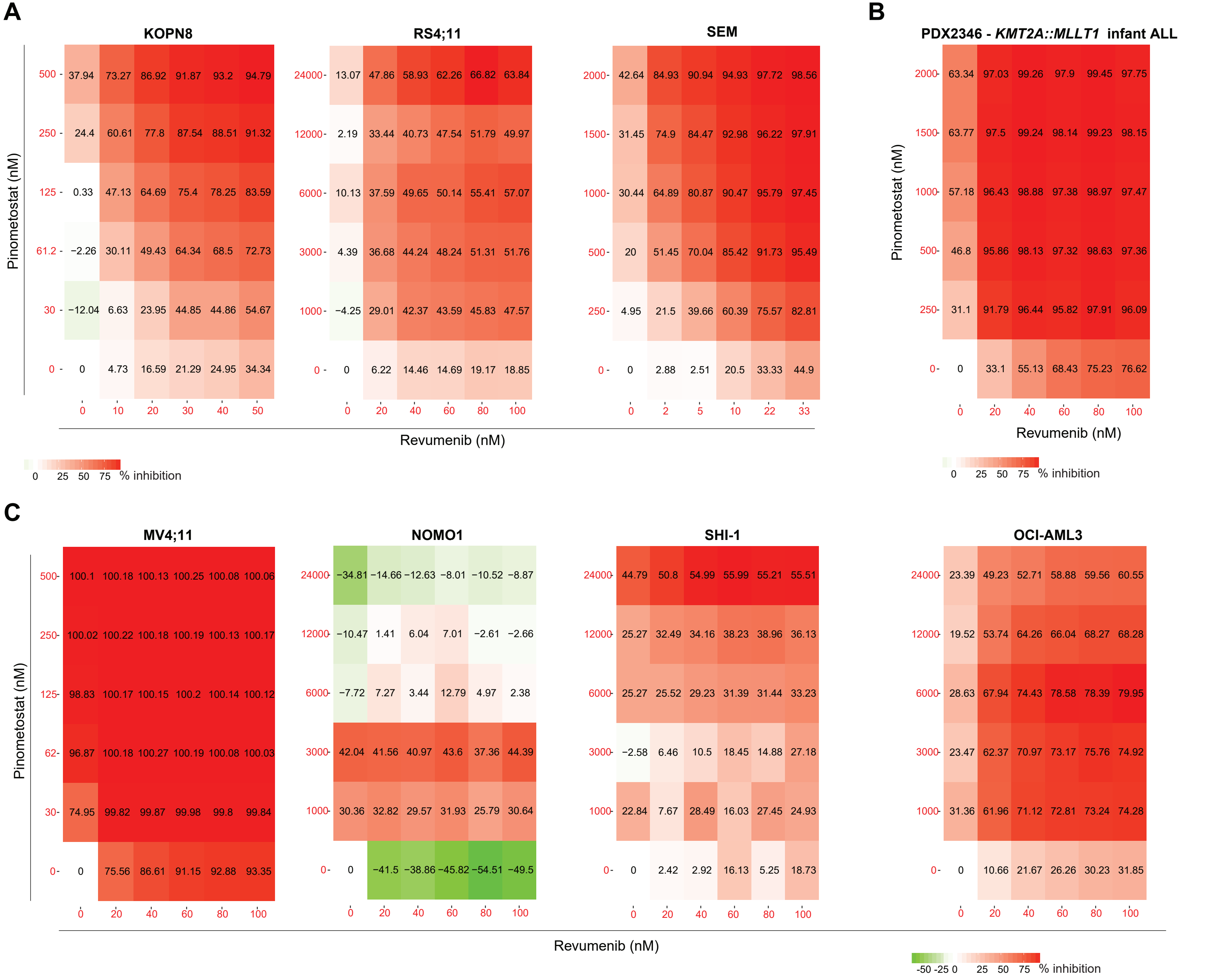

Supplementary Figure S3. Synergy between revumenib and pinometostat in *KMT2A*-rearranged ALL.
(**A**) Dose-response matrixes showing the percentage of inhibition compared to untreated cellsfor the indicated concentrations of revumenib (x-axis) and pinometostat (y-axis) as determined by 6 day pinometostat pre-treated cells followed by 4-day MTT assays of revumenib exposures in (A) KMT2A-rearranged ALL cell lines , (**B**) a PDX-derived KMT2A-rearranged infant ALL patient sample, and(**C**) KMT2A-rearranged AML cell line models. All cell line experiments are performed as biological duplicates (conducted as technical duplicates). Numbers in each box indicate the per-centage of inhibition compared to the DMSO controls. .
